# Assessing Morbidity and Outcomes of Posterior Lumbar Fusion in Elderly Patients: A Systematic Review and Meta-Analysis

**DOI:** 10.7759/cureus.81959

**Published:** 2025-04-09

**Authors:** Yeswanth Akula, Srinath Pammi, Sameer Rathore, Sonu Mehta, Pranshu Agrawal, George W Monaghan, Zhanzhe Zhang, Ajay Asokan, Maheswara Akula

**Affiliations:** 1 Department of General Surgery, East of England NHS Deanery, Cambridge, GBR; 2 Department of Trauma and Orthopaedics, Basildon University Hospital, Basildon, GBR; 3 Department of Trauma and Orthopaedics, Royal National Orthopaedic Hospital, London, GBR; 4 Department of Trauma and Orthopaedics, Airedale General Hospital, Keighley, GBR; 5 Department of Trauma and Orthopaedics, Wirral University Hospital, Birkenhead, GBR; 6 Department of Trauma and Orthopaedics, University College London, London, GBR; 7 Department of Trauma and Orthopaedics, The Royal London Hospital, London, GBR; 8 Department of Trauma and Orthopaedics - Spine Surgery, Basildon University Hospital, Basildon, GBR

**Keywords:** degenerative lumbar disorder, elderly population, functional and clinical outcome, lower back pain (lbp), morbidity and mortality, posterior lumbar fusion

## Abstract

This review aims to evaluate the morbidity, mortality, and functional outcomes associated with instrumented posterior lumbar arthrodesis in elderly patients aged 65 years and above. A systematic review and meta-analysis adhering to Preferred Reporting Items for Systematic Reviews and Meta-Analyses (PRISMA) guidelines was conducted using PubMed/MEDLINE (Medical Literature Analysis and Retrieval System Online), Cochrane, Embase, Emcare, CINAHL (Cumulated Index to Nursing and Allied Health Literature), and the international prospective register of systematic reviews (PROSPERO) databases. Relevant studies were identified based on inclusion criteria, with data extraction focusing on mortality rates, operative duration, blood loss, length of hospital stay, complication rates, and clinical and functional outcomes. Functional assessments included the Oswestry Disability Index (ODI), Japanese Orthopaedic Association (JOA) scores, and the Visual Analogue Scale (VAS) for pain intensity. Out of 6,241 studies, 45 met the inclusion criteria. The pooled mortality rate was 0.7% (95%CI: 0.2-1.3%), with a mean operative duration of 181.6 minutes (95%CI: 138.8-224.5 minutes) and an average blood loss of 337.8 ml. Complication rates included dural tears (4.3%), neurological deficits (2.2%), metalwork failure (7.06%), and wound dehiscence (3.29%). Significant postoperative functional improvements were observed, with reductions in ODI by 3.47 points, leg pain intensity by 4.29 points on the VAS, back pain intensity by 4.95 points on the VAS, and an increase in JOA scores by 9.9 points. Despite concerns regarding morbidity and mortality, this meta-analysis highlights that instrumented posterior lumbar arthrodesis in elderly patients is associated with considerable improvements in clinical and functional outcomes, supporting its role in the management of degenerative spinal conditions in this population.

## Introduction and background

As the global population ages, the management of lower back pain and sciatica stemming from degenerative lumbar disorders presents increasing challenges for surgeons and anaesthetists, both perioperatively and postoperatively [1]. Within the constraints of finite healthcare resources, policymakers may face pressures to allocate funds only to treatments demonstrating efficacy within specific patient cohorts, especially in the elderly who present with osteoporosis and multiple comorbidities, with the involvement of often more than one system [2].

Traditionally, degenerative lumbar disorders in elderly patients have been managed non-operatively. However, advancements in surgical and anaesthetic techniques have expanded the surgical indications within this demographic. Treatment modalities typically include simple decompression, discectomy, and internal stabilization, either anteriorly or posteriorly. Lumbar decompression with or without posterior fusion is among the commonly employed procedures for addressing these indications [3].

While numerous studies have established the efficacy of lumbar decompression in elderly populations [4], the role of lumbar fusion in this demographic remains contentious. Some studies have reported substantial complication rates, with major complications documented at 35% [5] and minor complications at 70% [6]. To address this controversy, we conducted a systematic review and meta-analysis to comprehensively evaluate the efficacy of posterior lumbar fusion in elderly patients.

## Review

Material & Methods

Search Strategy

The strategy adhered to the Preferred Reporting Items for Systematic Reviews and Meta-Analyses (PRISMA) guidelines [7]. A comprehensive electronic search was conducted across multiple databases, including PubMed/MEDLINE (Medical Literature Analysis and Retrieval System Online), Cochrane, Embase, Emcare, CINAHL (Cumulated Index to Nursing and Allied Health Literature), and the international prospective register of systematic reviews (PROSPERO) with no restrictions on publication dates. Additionally, reference lists of identified articles were manually reviewed to ensure the inclusion of relevant studies meeting the predetermined criteria. 

The inclusion criteria for this systematic review were defined to ensure the selection of relevant studies focusing on posterior lumbar fusion in elderly patients. Only studies involving patients aged 65 years and older were considered. The review included prospective and retrospective observational cohort studies that assessed the treatment of degenerative lumbar spinal disorders, such as spinal stenosis, spondylolisthesis, and degenerative disc disease, specifically in cohorts undergoing posterior instrumented fusion. To maintain data reliability and relevance, studies were required to have a minimum of 20 patients with extractable data specific to the instrumented fusion cohort. Additionally, selected studies needed to be matched with the population group and report at least one desirable outcome related to morbidity or clinical improvement.

Certain studies were excluded to minimize bias and maintain the specificity of the analysis. Database studies and case reports were not considered, as they often lacked detailed patient-level data. Studies focusing exclusively on a particular race, gender, or ethnic origin were excluded to ensure broader generalizability. Multicenter studies were not included due to variations in surgical techniques and institutional protocols. Furthermore, studies where data could not be clearly distinguished between simple decompression and fusion or where fusion techniques other than posterior were used were excluded. Studies involving infection cases, revision surgeries, and those evaluating non-primary fusion indications were also excluded. Finally, editorials, reviews, opinions, commentary articles, randomized controlled trials, systematic reviews, and meta-analyses were not included, as they do not provide the original patient data required for this analysis.

Selection Criteria

These were established through a rigorous process involving two independent reviewers who initially screened abstracts to identify articles eligible for full manuscript review. Subsequently, the full texts of selected articles were meticulously assessed by the same reviewers to determine their suitability for inclusion in the study. In instances where discrepancies arose, a third author intervened, and discussions were conducted until a consensus was achieved. Furthermore, a manual search of reference lists was conducted to ensure that relevant studies were not inadvertently overlooked. This comprehensive approach aimed to minimize bias and ensure the thorough inclusion of pertinent studies meeting the predetermined criteria.

Outcome Evaluation

Operative time, blood loss, length of hospitalization, and complications such as dural tears, nerve injuries, fractures, wound dehiscence, infections, myocardial infarction (MI), arrhythmias, pulmonary embolism (PE), deep venous thrombosis (DVT), urinary tract infections (UTI), cerebrovascular accidents (CVA), confusion, pneumonia, and ileus were evaluated. Functional outcomes included the Oswestry Disability Index (ODI), measured on a scale from 0% to 100%, with 0-20% indicating minimal disability and 80-100% indicating bed-bound status. Additionally, data on end-of-study back pain outcomes measured by the Visual Analogue Scale (VAS) and functional outcome data presented as Japanese Orthopaedic Association (JOA) scores were extracted and analysed.

Data Synthesis and Statistical Analysis

Meta-analyses were conducted independently for the following parameters: (i) Complication rates (dural tears, neuro defects, metalwork failure, wound dehiscence), (ii) Mortality, (iii) Mean operative duration, (iv) Mean estimated blood loss, (v) Mean length of hospitalization, (vi) Raw ODI difference, (vii) Raw VAS difference (leg), (viii) Raw VAS difference (back), and (ix) Raw JOA difference.

For all meta-analyses, outcomes were pooled by random effects with weights calculated by the inverse variance method [8]. Heterogeneity across trials was investigated by the Cochran Q test and measured by the I^2^ statistic, with I^2^ values exceeding 25%, 50%, and 75% indicating low, moderate, and high heterogeneity, respectively [9]. For continuous outcomes, only those studies with reported standard deviations were included in the calculations. Meta-analysis of VAS and ODI scores were summarized by the difference in means based on sample size and preoperative and postoperative means and standard deviations. A pre-post correlation of 0.5 was assumed. Meta-analysis was performed using R version 4.3.0 (R Foundation for Statistical Computing, Vienna, Austria).

Results

**Figure 1:**
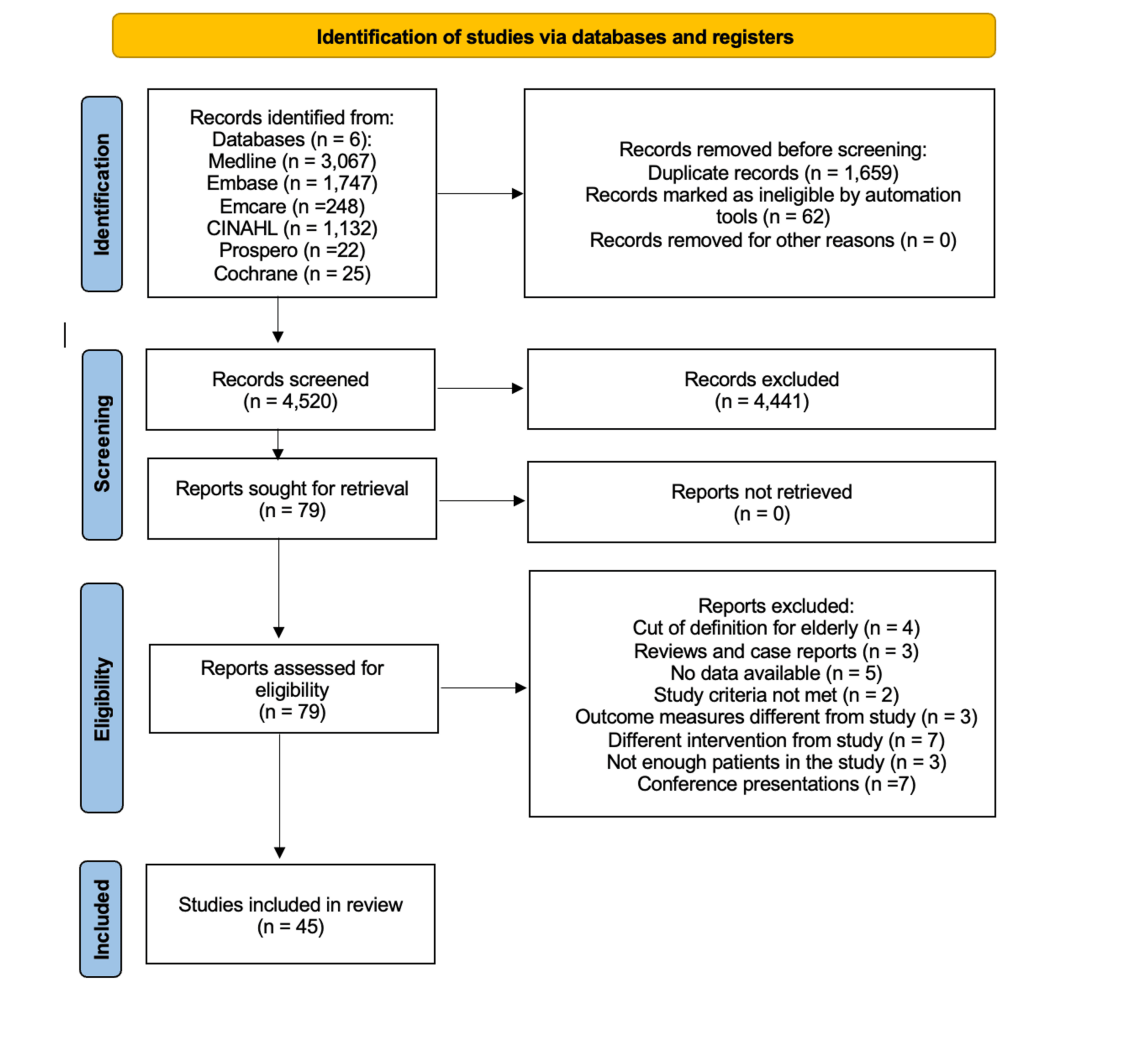
PRISMA flowchart for the database search and the included studies. PRISMA: Preferred Reporting Items for Systematic Reviews and Meta-Analyses

A total of 6241 studies were identified through our search strategy. After removing 1721 duplicate publications, 4520 articles underwent abstract and title screening yielding 79 articles, which underwent full-text analysis. Subsequently, 34 articles were excluded for various reasons, leaving 45 articles for analysis [8,10-53] (Figure 1).

Mortality

Mortality data was available from 13 studies [8,10,11,14,24,31,33,36,38,40,42,47,50] involving 127,723 patients. Rodgers et al. reported a mortality rate of 30% in patients with a mean age of 84 years who underwent open posterior lumbar interbody fusion (PLIF) within 18 months [54], while Liao et al. reported a mortality rate of 16% in their retrospective series [48]. 

Meta-analysis revealed an overall mortality rate of 0.7% (95%CI 0.2-1.3).

Operative Duration

Operative duration data from 25 studies [8,12-17,19-24,28,29,32,33,35,39,40,44-46,48,53] indicated a mean operative duration of 185 minutes (95% CI 108.2-261.8). 

Blood Loss and Hospital Stay

Perioperative blood loss data from 23 studies [12-18,20,21,23,24,29,32-35,39,40,44-46,48,53] showed a mean blood loss of 377 ml (95%CI 146-608). The mean length of hospital stay, based on 30 studies [8,10,12,14,15,18-24,28-35,37,39,40,44-48,50,53] was 8.2 days (95%CI 5.9-10.5).

Complications

Carreon et al. reported a 79.6% complication rate in elderly patients (>65 years) undergoing posterior decompression and fusion, with 21.4% experiencing major complications and 50% having two or more complications [40]. This review revealed various systemic and spinal procedure-related complications. 

Systemic complications included arrhythmia (n=39, 3.1%; 95%CI 2.1-4.4), congestive heart failure (CHF) (n=10, 1.8%; 95%CI 1.1-3.3), pneumonia/respiratory distress (n=51, 3%; 95%CI 2.2-4.0), delirium (n=84, 6.5%; 95%CI 5.3-7.8), stroke (n=11, 2.8%; 95%CI 1.6-5.1), UTI (n=137, 6.7%; 95%CI 5.5-8.0), renal failure (n=18, 2.3%; 95%CI 1.4-3.5), deep venous thrombosis (DVT) (n=1872, 1.3%; 95%CI 0.8-2.3), PE (n=2357, 1.5%, 95%CI 0.8-2.8), ileus (n=1705, 5.1%, 95%CI 4.1-6.3), MI (3.5%, 95% CI 2.5 to 4.9, n=1232), and syndrome of inappropriate antidiuretic hormone secretion (SIADH) (1.6%, 95% CI 0.6 to 4.3, n=4). 

Spinal procedure-related complications, when pooled, from 19 studies [8,11-15,19,24,30,32-35,37-40,44,53] included dural tears 4.3% (95%CI 5.35-10.87), neurological defects 2.2% (95%CI 1.35-3.06), metalwork failure 7.06% (95%CI 0-14.60), wound dehiscence 3.29% (95%CI 2.29-4.29%). 

Other spinal procedure-related complications were not measured due to the insufficiency of studies reporting these. Another meta-analysis reported reoperation (19%; 95%CI 10-43, five studies, 119 patients), hardware failure during surgery (12%; 95%CI, 4-23, five studies, 93 patients), infection (9%; 95%CI, 2-12, eight studies, 319 patients), pseudarthrosis (9%; 95%CI, 4-22, six studies, 149 patients), and neurological deficit (12%; 95%CI 9-22, eight studies, 443 patients) as the most prevalent complications in elderly patients [55].

Clinical and Functional Outcomes

**Figure 2:**
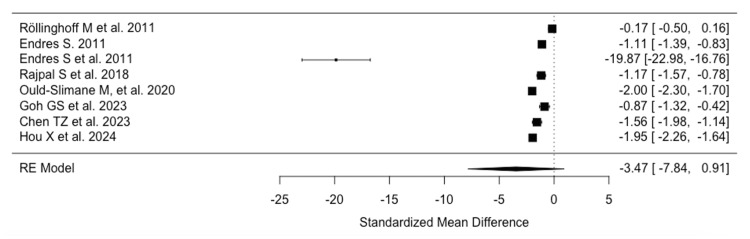
Raw Oswestry Disability Index difference References: [36,21,23,33,35,28,17,43]

Analysis of clinical and functional outcomes revealed significant improvements postoperatively. Meta-analysis of ODI scores from eight studies [17,21,23,28,33,35,36,43] (628 patients) showed a reduction in disability index by 3.47 (95%CI -0.91-7.83) (Figure 2). 

**Figure 3:**
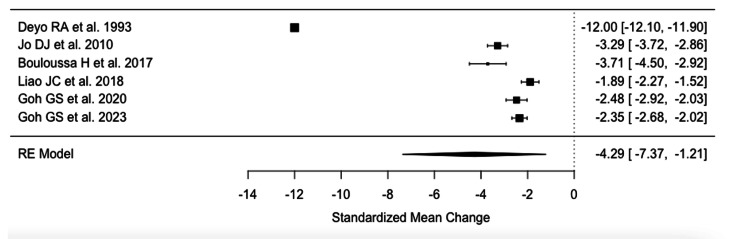
Raw Visual Analog Scale leg difference References: [31,30,24,48,19,28]

Meta-analysis of VAS scores for leg pain from six studies [19,24,28,30,31,48] (27567 patients) indicated a mean reduction in pain by 4.29 (95%CI 1.21-7.39) following fusion at final follow-up (Figure 3). 

**Figure 4:**
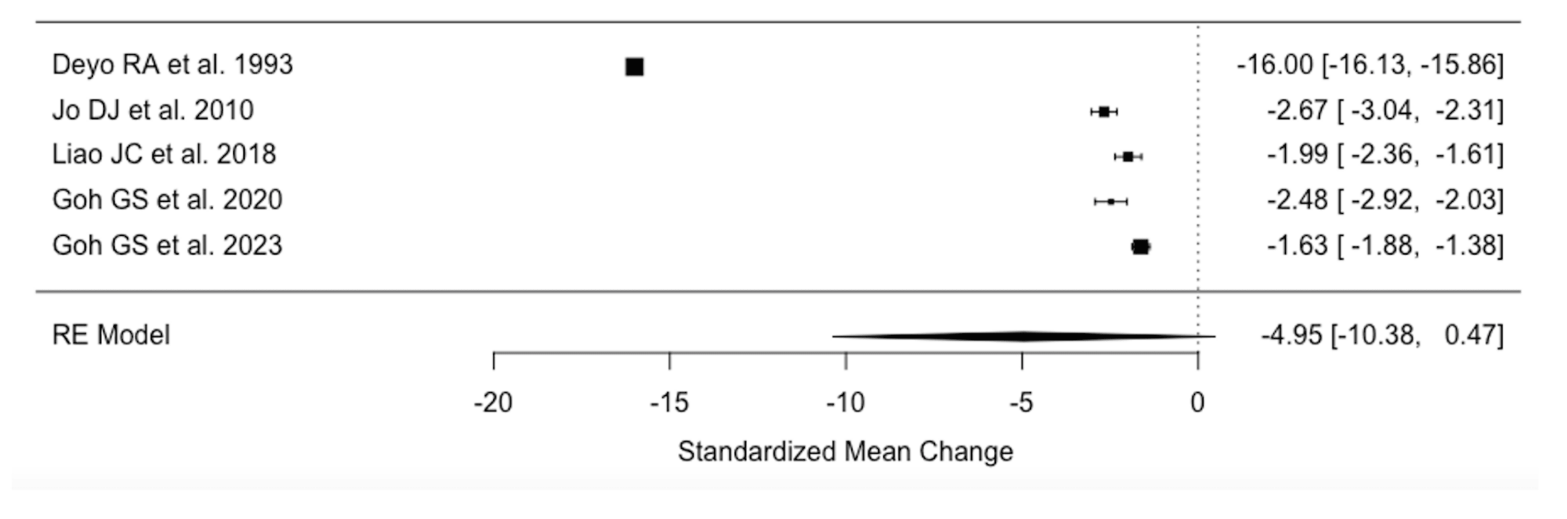
Raw Visual Analog Scale difference back pain References: [31,30,48,19,28]

Meta-analysis of VAS scores for back pain from five studies [19,28,30,31,48] (27,518 patients) indicated a mean reduction in pain by 4.95 (95%CI 0.47-10.38) following fusion at final follow-up (Figure 4).

Four studies have reported on the JOA score. Hayashi et al. observed a significant improvement in patients over 80 years of age, with the JOA score increasing from 9.3 preoperatively to 18.7 postoperatively [13]. Similarly, Onda et al. reported an improvement from 8.9 to 17.9 in cases involving instrumented fusion [38]. Okuda et al. documented a notable increase in the JOA score from 12 to 23 in patients above 70 years of age [49]. Chen et al. demonstrated substantial functional recovery, with one fusion group improving from 10.05 ± 1.40 to 25.40 ± 1.07 and another fusion group increasing from 9.90 ± 1.68 to 24.15 ± 0.81 at six months postoperatively [17].

Discussion

Degenerative lumbar spine disease presents a substantial clinical challenge, particularly within the elderly population, not only affecting their quality of life but also constituting a significant portion of community health expenditures [56]. The proportion of individuals over 65 years of age has doubled over the last two decades, representing more than 21% of the total population, and is projected to increase by 150% over the next 35 years, according to World Bank data [57]. Concurrently, the incidence of spinal fusion surgery has surged by 138.7% in this age group [58]. 

Unfortunately, discrepancies in the literature regarding mortality, morbidity, and clinical outcomes have created a notable gap in our collective understanding, fuelling ongoing debates over the justification of lumbar fusion in older adults [6]. Consequently, the risks and benefits of each aspect of surgical intervention must be meticulously evaluated, considering the complex array of factors that can impact outcomes.

Age, as a standalone factor, is widely recognized to correlate with increased rates of morbidity and mortality, a sentiment echoed by Deyo et al. [31]. In our review, meta-regression analysis affirmed a direct correlation between mortality rates and advancing age. 

Carreon et al. reported a substantial complication rate of 80% in elderly patients undergoing posterior decompression and arthrodesis, with 21% experiencing major complications, predominantly wound infections, and 70% experiencing at least one minor complication, commonly UTIs [40]. Raffo and Laureman observed a major complication rate of 35% in patients aged over 80 years, underscoring the association between comorbidity and major complications [59]. Conversely, Ragab et al. found no increased morbidity in patients aged 70 and older [60].

Balabaud et al. noted no correlation between complications and factors such as gender, American Society of Anesthesiologists (ASA) grading, BMI, anticoagulant use, comorbidity Charlson score, neurological deficit, laminectomy levels, or instrumented levels in patients over 80 [32]. However, they reported a high morbidity rate influenced by operative factors such as blood loss, operative time, instrumentation, previous surgery, and dural tears.

Incidental durotomies are common complications of lumbar fusion surgeries, with reported incidence rates ranging from 1% to 17%. Buck and Yoon reported an incidence rate of 4.65% in their retrospective database series [61].

The current review documented rates of incidental durotomies of 4.3% incidence rate, neurological deficits in 2.2% of cases, hardware failure in 7.06% of cases, wound dehiscence in 3.29% of cases, and common systemic complications such as UTIs and delirium, each with rates of 6.7% and 6.5%, respectively.

Despite these challenges, several studies in our review advocate for the efficacy of lumbar arthrodesis in the elderly population [62]. Meta-analyses of clinical and functional outcomes consistently demonstrate substantial improvements in disability, pain, and functional status postoperatively, suggesting the merit of considering such surgeries in this demographic [63]. Cassinelli et al. [44] emphasized that age, instrumentation, or comorbidities should not preclude these procedures, a sentiment echoed by Okuda et al. [49], who reported satisfactory outcomes for posterior lumbar interbody fusion (PLIF) in elderly patients.

Long-term outcomes, including adjacent segment degeneration and delayed union, were studied by various authors, with most concluding that perioperative complications did not adversely affect long-term clinical outcomes in elderly patients [12,13,63,64]. However, Hayashi et al. noted lower bony union rates and worsened clinical outcomes due to postoperative osteoporosis-related vertebral fractures in patients over 80 years of age [13].

Our review is constrained by a lack of cost analysis, primarily due to insufficient data. Future studies exploring cost considerations would provide valuable insights for policymakers and further validate the role of lumbar fusion in elderly patients.

## Conclusions

Degenerative lumbar spinal surgery in the elderly remains a contentious issue. Our review highlights the high rates of morbidity and mortality in this population, which correlate directly with advancing age. However, while increased complication rates do not necessarily translate to worsened clinical outcomes, our findings underscore significant improvements in disability, pain intensity, and function following fusion surgery in elderly patients. Surgeons and anaesthetists must carefully weigh the complexities of such procedures with their patients, but the notable clinical benefits observed in our review advocate for considering surgery in elderly populations. Our study contributes to the existing literature, providing valuable insights for peri- and post-operative care planning in this challenging demographic. Future studies exploring cost considerations, evaluating preoperative criteria for elderly patients would be valuable.
